# An international comparison of retinopathy of prematurity grading performance within the Benefits of Oxygen Saturation Targeting II trials

**DOI:** 10.1038/eye.2017.150

**Published:** 2017-07-28

**Authors:** B W Fleck, C Williams, E Juszczak, K Cocker, B J Stenson, B A Darlow, S Dai, G A Gole, G E Quinn, D K Wallace, A Ells, S Carden, L Butler, D Clark, J Elder, C Wilson, S Biswas, A Shafiq, A King, P Brocklehurst, A R Fielder, BOOST II Retinal Image Digital Analysis (RIDA) Group, David G Cottrell, Rasha Altaie, Rohan W Essex, Geoffrey C Lam, Michael Forrest, Shaheen Shah, James Smith, Jeremy Smith, Deepa Taranath, Michael O'Keefe

**Affiliations:** 1 Department of Child Life and Health, University of Edinburgh, Edinburgh, UK; 2 Department of Paediatric Ophthalmology, School of Social and Community Medicine, University of Bristol, Bristol, UK; 3 Clinical Trials Unit, National Perinatal Epidemiology Unit, University of Oxford, Oxford, UK; 4 Department of Ophthalmology, Princess Alexandra Eye Pavilion, Edinburgh, UK; 5 Neonatal Unit, Royal Infirmary of Edinburgh, Edinburgh, UK; 6 Cure Kids Professor of Paediatric Research, Department of Paediatrics, University of Otago, Christchurch, New Zealand; 7 Department of Paediatric Ophthalmology, Starship Children’s Hospital, University of Auckland, Auckland, New Zealand; 8 Department of Ophthalmology, University of Queensland, Brisbane, Australia; 9 Department of Pediatric Ophthalmology, Children’s Hospital of Philadelphia, Philadelphia, USA; 10 Department of Pediatric Ophthalmology, Duke University, Durham, USA; 11 Department of Ophthalmology, University of Calgary, Alberta, Canada; 12 Department of Ophthalmology, Royal Children’s Hospital, Victoria, Australia; 13 Department of Paediatric Ophthalmology, Birmingham and Midlands Eye Centre, Birmingham, UK; 14 Department of Ophthalmology, Aintree University Hospital, Liverpool, UK; 15 Department of Ophthalmology, Royal Children’s Hospital, Melbourne, Australia; 16 Department of Paediatrics, University of Melbourne, Melbourne, Australia; 17 Department of Ophthalmology, Chelsea and Westminster Hospital, London, UK; 18 Department of Paediatric Ophthalmology, Manchester Royal Eye Hospital, Central Manchester Foundation Trust and Manchester Academic Health Sciences Centre, Manchester, UK; 19 Department of Ophthalmology, Newcastle Eye Centre, Newcastle, UK; 20 Department of Ophthalmology, Division of Optometry and Visual Sciences, City University, London, UK

## Abstract

**Purpose:**

To investigate whether the observed international differences in retinopathy of prematurity (ROP) treatment rates within the Benefits of Oxygen Saturation Targeting (BOOST) II trials might have been caused by international variation in ROP disease grading.

**Methods:**

Groups of BOOST II trial ophthalmologists in UK, Australia, and New Zealand (ANZ), and an international reference group (INT) used a web based system to grade a selection of RetCam images of ROP acquired during the BOOST II UK trial. Rates of decisions to treat, plus disease grading, ROP stage grading, ROP zone grading, inter-observer variation within groups and intra-observer variation within groups were measured.

**Results:**

Forty-two eye examinations were graded. UK ophthalmologists diagnosed treat-requiring ROP more frequently than ANZ ophthalmologists, 13.9 (3.49) compared to 9.4 (4.46) eye examinations, *P*=0.038. UK ophthalmologists diagnosed plus disease more frequently than ANZ ophthalmologists, 14.1 (6.23) compared to 8.5 (3.24) eye examinations, *P*=0.021. ANZ ophthalmologists diagnosed stage 2 ROP more frequently than UK ophthalmologists, 20.2 (5.8) compared to 12.7 (7.1) eye examinations, *P*=0.026. There were no other significant differences in the grading of ROP stage or zone. Inter-observer variation was higher within the UK group than within the ANZ group. Intra-observer variation was low in both groups.

**Conclusions:**

We have found evidence of international variation in the diagnosis of treatment-requiring ROP. Improved standardisation of the diagnosis of treatment-requiring ROP is required. Measures might include improved training in the grading of ROP, using an international approach, and further development of ROP image analysis software.

## Introduction

Retinopathy of prematurity (ROP) is graded using the International Classification of Retinopathy of Prematurity (ICROP).^1^ While standard images are provided in ICROP, examiners must use subjective judgement when describing ROP in an infant. Variation in the rates of severe ROP between clinical centres have been attributed in part to observer bias.^2^ A number of studies have demonstrated inter-observer variation when grading ROP using retinal images.^3, 4, 5, 6, 7, 8, 9, 10^

Five international, multicentre randomised controlled trials of oxygen saturation targeting in very premature infants have recently been reported. The trial protocols were prospectively aligned to facilitate meta-analysis, the NeOProM collaboration.^11^ The trials were performed in UK,^12, 13^ Australia,^12, 13^ New Zealand,^12, 14^ Canada,^15^ and USA.^16, 17^

The Benefits of Oxygen Saturation Targeting (BOOST) II trials performed in UK, Australia, and New Zealand reported outcomes at the time of hospital discharge in 2013.^12^ While the participants (premature infants) were broadly similar across countries, a large difference in ROP treatment rates was noted.^12^ 153/798 (19.2%) of enroled infants were treated in the UK, compared to 75/975 (7.7%) in Australia and 23/306 (7.5%) in New Zealand.^12^ All ophthalmologists in the BOOST II trials were instructed to base their decision to treat on the ETROP^18^ definition of Type 1 ROP, however subjective interpretation of ROP disease signs may have varied between countries.

Within the BOOST II UK trial, ophthalmologists in 12 of the 34 trial centres used RetCam imaging (Natus Medical, Pleasanton, CA, USA) for ROP screening.^19^ Imaging was not performed in the other UK centres. These images gave us the opportunity to compare ROP grading decisions made by ophthalmologists in the UK, Australia, and New Zealand who participated in the BOOST II trials. An international reference group was used as the gold standard. We aimed to determine whether international variation in the interpretation of images and subsequent treatment decisions was present, evidenced in our opportunistic cohort.

## Materials and methods

### Ophthalmologists participating in the BOOST II trials

Within the BOOST II trials, local ophthalmology services for routine ROP screening and treatment were used. In the UK, BOOST II UK trial ophthalmologists were asked to attend a training session on ROP classification, and were provided with printed training materials. In Australia and New Zealand, all BOOST II trial ophthalmologists were asked to self-certify prior to the trials, using a training and assessment website http://www.boostnz.info/ROP/.

### Readers

Nine readers from Australia, two from New Zealand, and seven from UK who participated in the BOOST II trials were used (Supplementary Information). The groups from Australia and New Zealand were combined (ANZ) because the number of readers from New Zealand was low, and because ophthalmologists in Australia and New Zealand have a close working relationship for training and clinical practice. An international reference group of six experienced ophthalmologists with an interest in ROP who had not participated in the trials was used as the ‘gold standard’ (INT) (Supplementary Information). The international reference readers were from UK (2), USA (2), Canada (1), and Australia (1). The median (range) number of year’s experience of the readers in performing clinical ROP screening examinations was 25 (14–26) for the UK group, 15 (3.5–40) for ANZ, and 21 (10–38) for the international reference group.

### Reading experiments

Each reader logged on to the study website and was given detailed instructions on how to classify the study images. Readers were referred to ICROP,^1^ but standard comparison images were not given. To protect patient anonymity, no clinical data were provided. For each eye examination, drop down menus were used to grade ROP, and a decision to ‘treat’ or ‘not treat’. The order of eye examinations was randomised each time a reader logged on. On completion of grading, data were downloaded to an Excel spreadsheet for analysis.

### Eye examination images

Images were selected by the lead study ophthalmologist (BWF) for high image quality and readability. An eye ‘examination’ was a set of one to five images obtained when examining one eye of one infant on one occasion. All selected eye examinations were performed prior to treatment. Forty-two eye examinations obtained from six centres were used (Supplementary Information). In some infants more than one eye examination was used, to ensure a range of ROP disease severity was available for review. When more than one examination was used from the same infant, each examination was performed on a different date. In 31 infants one eye examination was used, in 3 infants two examinations were used, and in one infant five examinations were used. Six of the 42 eye examinations, illustrating a range of ROP severity, were duplicated to allow measurement of intra-observer variation. Each reader assessed 48 eye examinations. Seventeen of the 42 (40.5%) image sets were obtained at the time when a decision to treat was made, or immediately prior to treatment. Thirteen of the 42 (31.0%) image sets were from infants who did not require treatment at the time of imaging, but who were subsequently treated. Twelve of the 42 (28.6%) image sets were from infants who were not treated for ROP at any time.

### Infants

RetCam images from 35 infants were used, linked to clinical data from the BOOST II UK trial. Thirty-four infants were white, one was British Pakistani. Seventeen (48.6%) infants were female and 18 were male. The mean gestational age was 25^+2^ weeks, range 22^+6^–27^+6^ weeks. The mean (SD) birth weight was 785 (170) g, range 366–1115 g. Twenty-three of the 35 (65.7%) infants were treated for ROP at some time in their clinical course.

### Statistical analysis

Descriptive statistics were used to summarise data according to type and distribution using counts/percentages for categorical data, means (standard deviations [SD]) for normally distributed continuous variables, and medians (ranges) for other continuous variables. Data obtained from duplicate eye examinations were only used for the calculation of intra-observer variation and were excluded from all other analyses. Inter-observer variation (Fleiss kappa) and intra-observer (Cohen kappa) values were calculated using the online tool www.statstodo.com. Conventionally, a kappa of 0.2 or less is considered poor agreement, 0.21–0.4 fair, 0.41–0.6 moderate, 0.61–0.8 strong and more than 0.8 near complete agreement.^20^ These terms were used when reporting our results.

## Results

### Treatment decisions

Of the 42 eye examinations reviewed the mean (SD) number of examinations per reader judged to require treatment was 13.9 (3.49) for UK readers, 9.4 (4.46) for ANZ readers, and 12.8 (5.49) for the international readers. The difference between UK and ANZ readers was significant (*t*-test *P*=0.038, mean difference=4.49, 95% CI=0.27–8.72).

### Plus disease

Of the 42 eye examinations reviewed the mean (SD) number of examinations per reader judged as ‘plus’ disease was 14.1 (6.23) for UK readers, 8.5 (3.24) for ANZ readers, and 13.2 (6.31) for the international readers (Table 1). The difference between UK and ANZ readers was significant (*t*-test *P*=0.021, mean difference=5.69, 95% CI=0.98–10.40).

### Stage of retinopathy of prematurity

For each reader, the number of examinations read for each ROP stage was calculated. The mean (SD) for each reader group is given in Table 2. The mean number of eye examinations per reader classified as stage 2 was higher in the ANZ group than in the UK group (*t*-test, *P*=0.026, mean difference=7.47, 95% CI=1.00–13.94). For stage 3 there were no significant differences between the groups.

### Zone

For each reader, the number of examinations read for each ROP zone was calculated. The mean (SD) for each reader group is given in Table 3. The proportion of eye examinations read as each zone was not significantly different between any pair of groups.

### Inter-observer variation

Inter-observer variation Fleiss kappa measures for each classification variable are given in Table 4. Inter-observer agreement for the whole group of readers was ‘fair’ or ‘moderate’ for all measures. Agreement was highest within the ANZ group for all measures, with ‘moderate’ agreement for treatment decisions and for plus disease categories. Agreement was ‘fair’ for treatment decisions within the UK group. Agreement was poor for most measures within the INT group.

### Intra-observer variation

We measured intra-observer variation by including six duplicate examinations within the 48 eye examinations shown to each reader. The results are shown in Table 5. All kappa values were within the ‘strong’ or ‘near perfect’ agreement categories.

## Discussion

We have compared the ROP grading decisions of BOOST II trial ophthalmologists in UK with those in Australia and New Zealand. UK ophthalmologists demonstrated a lower threshold to treat than Australian and New Zealand ophthalmologists. UK ophthalmologists graded more images as plus disease, and more images as treatment-requiring. There were no significant differences in grading stage 3 disease or ROP zone. The UK ophthalmologists had more inter-observer variation than the Australian and New Zealand ophthalmologists. Intra-observer consistency appeared to be good among all ophthalmologists. The international reference ophthalmologists graded in a similar way to the UK ophthalmologists.

There were a number of limitations in our study. While the data were obtained within the context of a clinical trial, RetCam images and ROP clinical data were obtained from routine clinical screening examinations. RetCam imaging was used in a limited number of centres during the BOOST II UK trial, and in some centres was only used immediately prior to treatment. The quality of images obtained was variable. The completeness of accompanying clinical data from the treating ophthalmologists was variable. The set of RetCam images used for the study was selected, not random. The groups of readers from each country were biased towards experienced, research-active ophthalmologists. The international reference group was limited in number, and may not have been truly representative of broad-based international expertise. The sample size of both RetCam images and of readers was small and therefore of insufficient power to detect all but the largest differences.

The context of this study was a group of five oxygen trials in premature infants—the NeOProM collaboration.^11^ Significant differences in ROP treatment rates between countries were evident. Within the BOOST II trials performed in UK, Australia, and New Zealand, 153/798 (19.2%) of enroled infants were treated in the UK, compared to 75/975 (7.7%) in Australia and 23/306 (7.5%) in New Zealand.^12^ Thus, in the UK 153 infants were treated, and 645 were not treated. In Australia and New Zealand combined (ANZ), 98 were treated and 1183 were not treated. The difference in treatment rates was significant (Chi squared test *P*<0.0001, odds ratio=2.51, 95% CI=1.98–3.18). In the Canadian COT trial, 130/1003 (13%) of trial survivors at 36 weeks postmenstrual age had undergone ROP treatment or had Stage 4 or 5 ROP.^15^ In the American SUPPORT trial, 120/913 (13.1%) of trial survivors at 36 weeks postmenstrual age had undergone ROP treatment or had been diagnosed as having Type 1 ETROP.^18^ If the Canadian and USA trials are combined,^15, 16^ 250 of 1916 (13.0%) were treated. The difference in treatment rates between the North American trials and the ANZ trial was significant (Chi squared test *P*<0.0001, odds ratio=1.71, 95% CI=1.37–2.13), and the difference in treatment rates between the UK trial and the North American trials was also significant (Chi squared test *P*<0.0001, odds ratio=1.47, 95% CI=1.22–1.77). These differences are unlikely to be due to chance.

The baseline clinical characteristics of infants enroled in the BOOST II UK, BOOST II Australia and the BOOST New Zealand trials were very similar.^12^ In addition, the measured oxygen treatments given to the infants in the trials were very similar, as were morbidity measures (other than treatment for ROP), and mortality.^12^ The cohorts enroled in the Canadian and USA trials^15, 16^ were also similar to those in the BOOST II trials. It is therefore unlikely that the difference in treatment rates between the individual studies was due to differences in the patient populations.

Different treatment rates could potentially result in different visual outcomes. The 2 year outcome data from the UK and Australian trials^13^ and from the New Zealand trial^14^ gave visual outcome data. In the UK 23 of 718 infants (3.2%), in Australia 5 of 911 infants (0.55%), and in New Zealand 1 of 340 infants (0.29%) had severe visual impairment. Additional information was available for the subgroup of UK infants treated with the revised oxygen algorithm.^13^ Eighteen of 551 (3.3%) had severe visual impairment. Four of these had retinal detachment, 12 had cerebral visual impairment and in two the cause was not recorded. Thus 4 of 551 (0.73%) had severe visual impairment due to retinal detachment. The lower treatment rate in ANZ did not result in a higher rate of severe visual impairment.

Differences in ROP treatment rates have been documented between centres,^2, 21^ between countries,^22, 23^ and over time.^9, 24, 25, 26^ Some variation may be due to differences in the clinical characteristics of the populations under study, and to neonatal care practices. This is likely to be the case when comparing countries with differing health service characteristics and over periods of time.^9, 22, 24, 25^ The clinical characteristics of the infants in the BOOST II trials were very similar.^12, 13^ In this study, we have explored the possible contribution to the observed different rates of ROP treatment of international variation in disease grading.^2, 9^ Our results suggest such variation was present.

While inter-observer agreement for plus disease grading was ‘moderate’ within the ANZ group, it was ‘poor’ for the UK group. Previous studies have also found limited agreement between experts in the diagnosis of treatment-requiring ROP,^10, 27^ and of plus disease.^10^ Gschliesser found moderate inter-observer agreement (kappa 0.41) for the necessity for treatment, and ‘fair’ agreement (kappa 0.32) for plus disease.^10^ Chiang found ‘fair’ and ‘moderate’ weighted kappa agreement for the diagnosis of plus disease when each of a group of experts was compared to all the other experts in the group.^5^

While standardisation of ROP diagnostic grading may be approached by improved training of screening ophthalmologists,^28, 29^ an international approach is needed. Tools such as online training and assessment websites may be used. In Australia and New Zealand all BOOST II study ophthalmologists were asked to self-certify prior to the trials, using http://www.boostnz.info/ROP/.

The key component in ROP treatment decisions is the detection of plus disease, as defined by ICROP.^1^ Our study, and a number of other studies,^5^ show the limitations of clinical judgement based on reference photographs. As has occurred in diabetic retinopathy screening, a move towards the use of retinal images rather than clinical examinations is a prerequisite for the standardisation of diagnostic decisions.^30, 31^ Computerised image analysis techniques, trained by clinical experts, are needed to improve the objectivity of treatment decisions.^30, 31, 32, 33, 34, 35, 36, 37, 38, 39^

The planning of international ROP treatment trials requires improved training and standardisation of observers. In the Cryotherapy for ROP study, a second examiner was required to examine each infant within 3 days of the primary examiner, to confirm the presence of treatment-requiring ‘threshold’ disease.^40^ In 12% of cases, the two examiners disagreed on the presence of plus disease.^27, 40^ Ideally, retinal images should be used in trials, with central reading centres.^41^ Both clinical trials and clinical practice will benefit from the use of image analysis software that quantifies plus disease.

We found international variation in the diagnosis of treatment-requiring ROP. While excessively low rates of ROP treatments risk blindness, excessively high rates of ROP treatments should also be avoided. Treatment is invasive, and carries risks of ocular and systemic morbidity. Improved standardisation of treatment decisions is an important goal. Approaches might include the use of internationally standardised online training tools, and the development of image analysis software to quantify ROP plus disease.


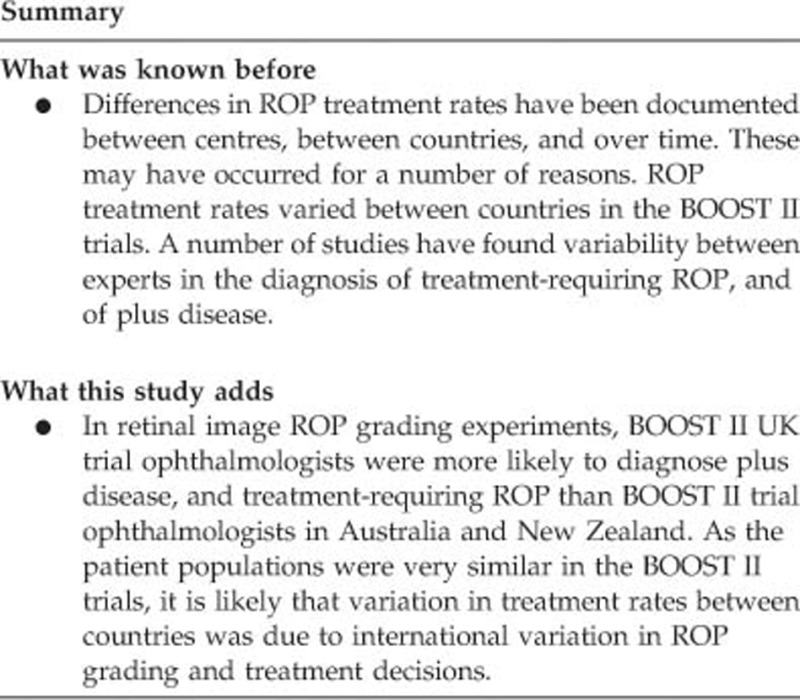


## Figures and Tables

**Table 1:** The mean (SD) number of eye examinations per reader classified as plus disease by reader group (*N*=42)

*Reader group*	*Plus disease*	*Pre-Plus disease*	*No Plus disease*	*Unable to assess*
UK	14.1 (6.2)	15.3 (5.8)	12.1 (7.6)	0.4 (0.5)
ANZ	8.5 (3.2)	13.6 (5.6)	19.6 (6.2)	0.3 (0.7)
INT	13.2 (6.3)	13.5 (1.8)	15.2 (6.2)	0.00

**Table 2:** The mean (SD) number of eye examinations per reader classified as each stage of ROP by reader group (*N*=42)

*Group*	*No ROP*	*Stage 1*	*Stage 2*	*Stage 3*	*AP-ROP*	*Unclassified*
UK	2.6 (1.1)	5.7 (2.9)	12.7 (7.1)	13.9 (4.0)	3.0 (1.6)	4.0 (3.1)
ANZ	2.6 (0.7)	3.8 (3.0)	20.2 (5.8)	11.0 (3.9)	2.0 (1.1)	2.3 (3.1)
INT	3.0 (0.6)	1.7 (2.4)	16.8 (2.5)	15.0 (4.3)	2.2 (1.6)	3.3 (4.8)

**Table 3:** The mean (SD) number of eye examinations per reader assessed for each ROP zone by reader group (*N*=42)

*Reader group*	*Zone I*	*Zone II*	*Zone III*	*Unclassified*
UK	5.9 (3.8)	28.7 (4.7)	7.0 (4.4)	1.3 (1.7)
ANZ	4.0 (3.5)	31.0 (4.5)	4.7 (3.7)	1.7 (1.7)
INT	6.0 (4.7)	26.0 (7.9)	6.4 (5.2)	2.5 (1.9)

**Table 4:** Inter-observer variation kappa statistics

*Group*	*Treatment decisions*	*Plus disease*	*ROP stage*	*ROP zone*
UK	0.33	0.2	0.25	0.12
ANZ	0.45	0.45	0.35	0.27
INT	0.19	0.18	0.27	0.13
All	0.35	0.3	0.31	0.22

**Table 5:** Intra-observer variation weighted Cohen kappa statistics

*Measure*	*Treatment decisions*	*Plus disease*	*ROP stage*	*ROP zone*
UK	0.95	0.84	0.92	0.74
ANZ	0.91	0.81	0.84	0.63
INT	0.72	0.91	0.9	0.88
All	0.88	0.85	0.88	0.83
