# Supplementary material for: An international comparison of retinopathy of prematurity grading performance within the Benefits of Oxygen Saturation Targeting II trials
Source: Eye (Lond). 2017 Jul 28;32(1):74–80. doi: 10.1038/eye.2017.150 (PMC5669461; doi:10.1038/eye.2017.150)
Supplement: Supplementary Information [file eye2017150x1.docx]

Supplementary Information

Trial registration numbers.

BOOST II Current Controlled Trials number: ISRCTN00842661; Australian New Zealand Clinical Trials Registry numbers: ACTRN12605000055606 and ACTRN12605000253606.

Writing group: all authors.

Collaborators: as listed in Acknowledgements.

The readers

UK trial ophthalmologists: Susmito Biswas, Lucilla Butler, David Clark, David G Cottrell, Brian W Fleck, Ayad Shafiq, Cathy Williams.

New Zealand trial ophthalmologists: Rasha Altaie, Shuan Dai

Australia trial ophthalmologists: James Elder, Rohan W Essex, Glen A Gole, Geoffrey C Lam, Michael Forrest, Shaheen Shah, Jeremy Smith, James Smith, Deepa Taranath

International expert group (not in trial): Susan M Carden (Australia), Anna Ells (Canada), Alistair R Fielder (UK), Graham E Quinn (USA), David K Wallace (USA), Clare Wilson (UK).

Images supplied by: Lucilla Butler, David Clark, Brian W Fleck, Michael O’Keefe, Ayad Shafiq, Cathy Williams.

#### NPEU Clinical Trials Unit, University of Oxford, UK

Peter Brocklehurst (Director and Chief Investigator), Ed Juszczak E (Medical statistician), Andy King (Analyst programmer).

Neonatologists

Ben J Stenson (UK), Brian A Darlow (New Zealand)
